# Comparison of Genetically Engineered Immunodeficient Animal Models for Nonclinical Testing of Stem Cell Therapies

**DOI:** 10.3390/pharmaceutics13020130

**Published:** 2021-01-20

**Authors:** Yoon-Young Kim, Jin-Soo Kim, Jeong-Hwan Che, Seung-Yup Ku, Byeong-Cheol Kang, Jun-Won Yun

**Affiliations:** 1 Department of Obstetrics and Gynecology, Seoul National University Hospital, Seoul 03080, Korea; yoonykim96@gmail.com (Y.-Y.K.); jyhsyk@snu.ac.kr (S.-Y.K.); 2 Department of Medical and Biological Sciences, The Catholic University of Korea, Bucheon 14662, Korea; js02ss04@hanmail.net; 3 Biomedical Center for Animal Resource and Development, Seoul National University College of Medicine, Seoul 03080, Korea; casache@snu.ac.kr; 4 Graduate School of Translational Medicine, Seoul National University College of Medicine, Seoul 03080, Korea

**Keywords:** immunodeficiency, severe combined immunodeficient (SCID) animal, animal species

## Abstract

For the recovery or replacement of dysfunctional cells and tissue—the goal of stem cell research—successful engraftment of transplanted cells and tissues are essential events. The event is largely dependent on the immune rejection of the recipient; therefore, the immunogenic evaluation of candidate cells or tissues in immunodeficient animals is important. Understanding the immunodeficient system can provide insights into the generation and use of immunodeficient animal models, presenting a unique system to explore the capabilities of the innate immune system. In this review, we summarize various immunodeficient animal model systems with different target genes as valuable tools for biomedical research. There have been numerous immunodeficient models developed by different gene defects, resulting in many different features in phenotype. More important, mice, rats, and other large animals exhibit very different immunological and physiological features in tissue and organs, including genetic background and a representation of human disease conditions. Therefore, the findings from this review may guide researchers to select the most appropriate immunodeficient strain, target gene, and animal species based on the research type, mutant gene effects, and similarity to human immunological features for stem cell research.

## 1. Introduction

Replacement of dysfunctional organs through transplantation is an attractive approach for the treatment of organ failure. However, the imbalance between supply and demand for replaceable human organs is a major problem for clinical transplantation. Although xenotransplantation may be an alternative option for this huge bottleneck, it is not a newly developed concept. The xenotransfusion of blood from lambs to humans in 1667 was first mentioned xenotransplantation in the context [1]. Clinical use of animal-originated organs has also been reported, that is, rabbit kidney transplantation to a human in 1905. Many kinds of animals are used for transplantation research, and this usage revealed the advantage and disadvantages of used animal models. Because of their closer phylogenetical relationship with humans, several trials involving the kidneys, hearts, and livers of nonhuman primates (NHPs) were conducted from the 1920s to 1990s [2,3]. Disadvantages of NHPs as animal models arise with continuous research, such as limited supplies due to the Convention on International Trade in Endangered Species of Wild Fauna and Flora (CITES), xenotransmission to humans, need for isolated facility and experts for the breeding [4]. Researchers have attempted to use pigs as the source animal for xenotransplantation since the 1990s, and the pig is currently considered the most appropriate candidate species. The pig’s relatively short maturation period, its size and physiological similarity to humans, the low-risk of xenozoonosis, and the application of genetic engineering techniques to produce porcine organs that are resistant to immunological rejection are the reasons for selecting the pig as a source animal [5]. The aforementioned advantages of pigs and NHPs revealed the necessity of the development of various genetically modified immunodeficient large animal models for preclinical xenotransplantation research, including severe combined immunodeficient (SCID), and resulted in considerable improvement of xenografts.

In addition to xenotransplantation, the value of the immunodeficient model has become increasingly important due to the emergence of regenerative medicine, including stem cell research. Although various types of stem cells are used as a cell source for cell-based therapy and regenerative medicine, immune rejection is a critical limitation in stem cell research. Therefore, an efficient immunodeficient model system is required to predict and analyze the therapeutic effect of stem cells after engraftment. Along with nude mice showing defective T cell-mediated immune responses [6], SCID mice can lack an adaptive immune response due to the deletion of the DNA-dependent protein kinase, catalytic subunit (PRKDC) gene [7] although innate immune cells, natural killer (NK) cells, and macrophages can reject the human cells transplanted in mice, thus significantly reducing the engraftment efficiency of human cells in SCID mice [8,9,10]. In contrast, NK cells do not develop in mice after the disruption of the interleukin 2 receptor subunit gamma (IL2RG) gene, thereby resolving this issue [11]. As immunodeficient mice, non-obese diabetic (NOD) mice possess a polymorphism in the inhibitory receptor signal regulatory protein alpha (SIRPA) gene, making it possible to bind to the human CD47, preventing macrophage-mediated rejection of human cells in these mice [8,12,13]. Transgenic expression of the human SIRPA gene in SCID mice also improves the efficiency of human cell engraftment [8,9,10]. NOD/SCIDIl2rg^−/−^ (NSG) mice are another example of SCID mice. NSG mice present an optimized transplantation model for human hematopoietic stem cells (HSCs) and serve as a suitable humanized mouse model to reconstitute the human immune system in vivo [11,12,13,14,15]. As such, different types of immunodeficient models can be utilized in biomedical research on stem cell or organ xenotransplantation and humanized model creation. In this review, we provide perspectives on the various immunodeficient model systems with different target genes, highlighting the advantages of a large animal model.

## 2. Immunodeficient Animals

### 2.1. Genes Involved in Immunodeficiency

#### 2.1.1. Forkhead Box N1 (FOXN1)

Functional deficiency of FOXN1 leads to a nude SCID animal. The “nude” phenotype, first identified in mice as a result of mutations in a single gene, originally named winged-helix nude (WHN), and recently termed as FOXN1 [16], encoding an essential transcription factor for the development and function of thymic epithelial cells [17,18,19]. The FOXN1 gene belongs to the forkhead box gene family, which comprises a diverse group of “winged-helix” transcription factors involved in aging, development, metabolism, and cancer [20]. Mice homozygous for the “nude” mutation are hairless, with retarded growth and lower fertility. The hairlessness is caused by the absence of free sulfhydryl groups in the midfollicle region [21,22,23,24]. The “nude” FOXN1 gene regulates the balance between proliferation and differentiation of keratinocytes in the hair follicle [25,26]. Male “nude” mice do not exhibit any motile sperms, whereas the females present low egg counts and small ovaries [24]. These mice present altered hormonal status, with changes in the serum levels of estradiol, progesterone, and thyroxine [27]. The thymus is the primary lymphoid organ for T cell differentiation and repertoire selection [28,29]. The thymus is absent at birth in these mice, and very few lymphocytes occur in the spleen and lymph nodes [30,31].

#### 2.1.2. DNA-Dependent Protein Kinase Catalytic Subunit (PRKDC)

PRKDC encoding a DNA-dependent protein kinase catalytic subunit (DNA-PKcs) is a critical component of the nonhomologous end-joining (NHEJ) pathway of the DNA double-strand breaks (DSBs) repair system. DSBs occur during predesigning processes, such as V(D)J (variable, diversity, and joining regions) recombination or class switch recombination that takes place during lymphocyte development [32,33,34,35]. Therefore, humans and several other mammals with defective PRKDC genes in NHEJ cannot undergo V(D)J recombination, which inhibits lymphocyte development and results in SCID characterized by an absence of functional T cells and B cells [36,37,38,39].

#### 2.1.3. Interleukin 2 Receptor Subunit Gamma (IL2RG)

IL2RG encodes the common gamma chain (γc) and is located on the X chromosome (Xq13). IL2RG gene is essential for interleukin signaling pathways, such as that of IL-2, IL-4, IL-7, IL-9, IL-15, and IL-21. These cytokines are critical for lymphocyte development and function, and they promote the regulation of T cell differentiation and peripheral tolerance. Furthermore, they increase the cytolytic activity of NK cells and B cell differentiation [40,41,42]. Mutations in γc cause X-linked SCID (X-SCID) in humans, which is characterized by profound defects in cellular and humoral immunity [43,44]. This gene also presents a similar working pattern in animals. Knockout of IL2RG resulted in the X-SCID phenotype in male pigs [45]. In classical X-SCID, defective IL2RG results in the deficiency of T and NK cells, or nonfunctional lymphocytes, and malfunctioning B lymphocytes (T-/B+/NK+SCID). Non-classical X-SCID, with a missense or potential non-loss of function mutation, is characterized by low numbers of T cells and normal numbers of NK and B cells (T-/B+/NK+SCID) [46,47,48].

#### 2.1.4. Recombination Activating Gene 1 and 2 (RAG1 and RAG2)

Two closely linked genes, RAG1 and RAG2, are essential for the somatic recombination process of the gene elements encoding the variable (V), diversity (D), and joining (J) segments, thereby generating a diverse repertoire of antigen-specific receptors on the surface of T and B lymphocytes [49]. Defects in the initiation of the V(D)J recombination process lead to a severe block or defective generation of T and B cells. SCID resulting from defects in RAG1 and RAG2 is characterized by severe depletion in mature T and B cell numbers, whereas NK cells are present in normal numbers (T-/B+/NK+SCID) [50,51]. This role of RAG1 and RAG2 has been demonstrated by gene targeting in mice. The phenotype of RAG1 and RAG2 knockout murine models is identical to that of the human condition, exhibiting severe and early blockade of both T cells and B cell development. This results in severe lymphopenia, with a virtual absence of T and B cells and the presence of circulating NK cells in the immunological phenotype.

#### 2.1.5. Janus Kinase 3 (JAK3)

The tyrosine kinase JAK3, belonging to the Janus family of kinases, plays a crucial role in the hematopoietic cytokine signaling pathway associated with γc [51,52], and its deficiency is associated with the absence of T lymphocytes and NK cells and the presence of nonfunctional B lymphocytes (T-/B+/NK+SCID) in humans, mimicking the abnormalities by γc mutations in X-SCID patients [53,54,55,56]. Genetic experiments in mice have shown that JAK3-mediated signaling is essential for lymphocyte development. Because of the close association between JAK3 and γc, a mutation in either protein results in the same clinical pathology and immunophenotypic characteristics. JAK3 knockout mice exhibit immune deficiency as demonstrated by the absence of T and NK cells and normal numbers of poorly functional B cells (T-/B+/NK+SCID) [53,57,58,59].

#### 2.1.6. Artemis (DCLRE1C)

The Artemis (DCLRE1C) gene encodes an endonuclease that cleaves the hairpins generated by the RAG1/RAG2 proteins [60]. Artemis deficiency is an autosomal recessive disorder that affects the mechanism of recombination of the T cell receptor and B cell receptor complexes [61]. It has been demonstrated that SCID pigs with a defect in the Artemis gene lack T and B lymphocytes but produce NK cells (T-/B+/NK+SCID) [62,63].

#### 2.1.7. Beta-2-Microglobulin (B2M) and Perforin 1

As a component of MHC class I molecules, B2M is related to the development of cytotoxic T cells and NK cell function [64]. Perforin encoded by the perforin gene is a pore-forming protein found in the granules of cytotoxic T lymphocytes and NK cells [65]. Kagi et al. reported [66] that the effects of T cells and NK cells are impaired in perforin-deficient mice.

#### 2.1.8. Adenosine Deaminase (ADA) and Adenylate Kinase 2 (AK2)

ADA is a key enzyme of the salvage pathways of purine metabolism. ADA deficiency can cause comprehensive lymphocyte apoptosis, leading to a SCID with severe T, NK, and B lymphocytopenia [67]. AK2 is an enzyme localized in the mitochondrial intermembrane space that plays a key role in the adenosine diphosphate generation [68]. AK2 deficiency, also known as reticular dysgenesis, displays SCID with the absence of T, B, and NK cells [69].

#### 2.1.9. Coronin-1A (CORO1A)

CORO1A, predominantly expressed in hematopoietic cells, is a highly conserved actin-binding protein that promotes F-actin disassembly. CORO1A deficient mice have reduced peripheral T cells due to increased apoptosis, causing T-/B+/NK+SCID [70].

### 2.2. Types and Characteristics of Immunodeficient Animals

#### 2.2.1. Nude Animals

The term “nude” refers to a lack of body fur. The first known immunocompromised mice were reported by Grist in 1962 (Ruchill Hospital, Glasgow, UK). Nude mice exhibited thymic aplasia and lacked T lymphocytes [24]. Since their discovery, the immunodeficiency of these animals has made them valuable hosts for xenografts, primarily for cancer research [71,72]. The nude mouse phenotype is caused by a mutation in the WHN gene, which encodes the FOXN1 transcription factor [16,21,23,73]. As a result, nude mice present defective adaptive immune responses, such as T cell-mediated immune responses requiring antibodies, and they exhibit leakage of T cells with age [6].

#### 2.2.2. SCID Animals

SCID, representing a severe form of primary immunodeficiencies, consists of diseases characterized by early blockage of T cell differentiation. Various forms of human SCID have been characterized and categorized according to inheritance, phenotype, and the involving genes [49]. Bosma et al. [36] first described SCID mice lacking both functional T and B lymphocytes as compared with T cell-deficient nude mice. PRKDC gene and RAG1/RAG2 mutation mouse were defined as a SCID mouse used widely in biomedical research. SCID mice were the first recipients of HSC and peripheral blood mononuclear cell (PBMC) transplants [74,75]. The engraftment efficiency of human tumors is higher in SCID mice than in nude mice [76]. However, the transplantation efficiency of human blood cells and tumor cells is not as high as expected, as the remnant NK cells prevent the homing and maintenance of human cells.

#### 2.2.3. SCID/Beige Animals

Since the beige mutation selectively impairs NK cell function, SCID/beige mice were developed by crossbreeding SCID and beige mice to overcome the effects of NK cells [77]. The SCID/beige mice exhibited severely reduced NK cell functions along with phagocytosis, characteristic of the beige mice, and T and B deficiency, characteristic of SCID mice [78]. The uptake rate of human tumor cells was higher in SCID/beige mice than in SCID mice [78]; however, the engraftment rate of human HSCs was not noticeably higher [79].

#### 2.2.4. NOD/SCID Animals

NOD mice with diabetes mellitus caused by the destruction of pancreatic islets by T lymphocytes were discovered in 1980 by Makino et al. [80]. NOD mice were reported to acquire multiple immune abnormalities, including complement loss and impaired NK, macrophage, and dendritic cell functions [81]. NOD/SCID mice were developed by crossbreeding NOD and SCID mice, and these mice did not develop diabetes because of functional T lymphocyte loss. Moreover, multiple defects in the innate and adaptive immunity of these mice make them better recipients for human HSC and solid tumor transplants [82].

#### 2.2.5. NOD/SCID-Based Immunocompromised Animals

Although NOD/SCID mice presented advanced and optimal properties, they exhibit some residual NK activities, and several attempts have been made to eliminate or suppress these properties for improved transplantation efficiency. These include crossbreeding with B2M- or perforin-deficient mice. B2M- [83] or perforin-null mice [66] crossed with NOD/SCID strain have been known to reduce innate immunity by preventing the functional activity of NK cells. The common chain (γc, CD132), also known as IL2RG, is a cytokine receptor subunit and represents the receptor complex for six different interleukin receptors, which are essential for lymphocyte and NK cell development, i.e., IL-2, IL-4, IL-7, IL-9, IL-15, and IL-21 [84]. NOD/SCID mice with complete loss of NK cells were developed by crossbreeding with IL-2 receptor-deficient mice, i.e., NOG and NSG (NOD/SCID/Il2rgnull: NOG [85], NOD/SCID/Il2rgnull: NSG [86]), or JAK3-deficient mice, i.e., NOJ (NOD/SCID/Jak3null) [85]. NOG mice present a NOD/ShiJic-Prkdcscid genotype with partial deficiency of IL2R [87], whereas NSG mice present a NOD/ShiSzJ-Prkdcscid genotype with complete deficiency of IL2R [75].

## 3. Comparison of Animal Species Used for Producing SCID Animals

SCID is defined as the lack or impairment of an adaptive immune system. It is primarily characterized by lymphopenia and a lack of thymocytes, absent or small thymus, and abnormalities in other immune tissues. SCID occurs spontaneously in humans, mice, horses, dogs, and pigs [36,39,88,89]. X-SCID animals, resulting from a defect in the γc of IL2RG located on the X chromosome, include rats [90], marmosets [91], pigs [45], and dogs with gene mutation [92]. Based on the fact that functional defects including T, B, and/or NK loss can lead to severe immunodeficiency, SCID conditions have been transgenically introduced into mice, rats, pigs, and NHPs to establish the research models providing insights into the mechanism of SCID and offer valuable tools for biomedical research including xenotransplantation of human stem cells [45,93,94,95,96,97]. Several mouse models of immunodeficiency have been reported, and mice are widely used in research due to their ease of handling, small size, and short lifespan. However, their small size complicates certain surgical procedures, such as those pertaining to eyes and blood vessels, resulting in inadequate long-term in vivo evaluation [98]. In contrast, large animals such as pigs and monkeys have larger organ size and longer lifespan and may therefore present alternative SCID models. The pig is a large animal model with more similarities with humans in terms of genetics, anatomy, and physiology. Their immune system presents an 80% resemblance with that of humans based on the analyzed parameters, compared with a human-to-mouse resemblance of only 10%. This confers an advantage of using pigs as an immunological model and in other biomedical research. However, they require advanced handling and a large-scale facility, resulting in high costs. Moreover, large animals exhibit longer sexual maturity and gestation periods with relatively long-term follow-up [99].

### 3.1. Types of Animals

#### 3.1.1. Mouse and Rat

**Figure 1:**
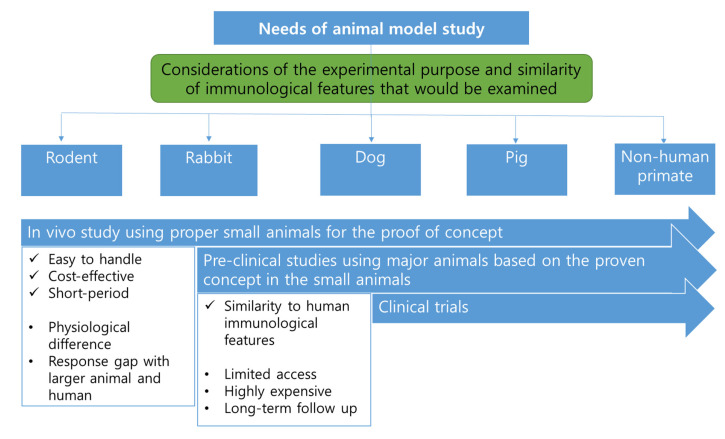
The need for animal study and selection of proper animals.

Mouse models are easy to handle and breed, and they are widely used because of their high reproductive ability (Figure 1). However, their immune system differs from that of humans [98]. Rats are phylogenetically similar to mice; however, they are more metabolically and physiologically similar to humans. Therefore, the rat is preferred to the mouse for modeling metabolic diseases and for use in physiological, pharmacokinetic, pharmacodynamic, and toxicological studies for preclinical efficacy and safety testing [100,101,102,103,104].

The rat is also the preferred animal model for stem cell therapy for neurological diseases and the evaluation of behavioral, psychological, and cognitive functions in response to drug treatment [102,105,106]. Stem cell-based therapies for heart diseases also employ rat models. Because of the differences in the heart rate of mice and humans, the mouse model is not suitable for evaluating therapeutic strategies for heart diseases. Rat models, with slower heart rates, are more suitable for such studies [107]. Rat models have been extensively used to evaluate the efficacy of human stem cell therapy for heart diseases, such as myocardial infarction and heart failure [107]. Another advantage of the rat model is their body size. Rats are significantly larger than mice, thus allowing more complicated surgical procedures for cell transplantation and providing greater blood supply. Rat models are more beneficial for testing the efficacy of stem cell-based therapy with less embolism or microvascular thrombosis. Therefore, the immunodeficient rat is comparable to the immunodeficient mouse for preclinical evaluation of the efficacy of human stem cell-based therapy.

#### 3.1.2. Rabbit

Rabbits belong to the family Leporidae of the order Lagomorpha and are phylogenetically closer to humans than rodents. They are very docile, easy to handle in animal facilities, and are relatively small-sized [108]. Various strains of rabbits are present, including the Himalayan and Dutch-belted; however, the New Zealand White rabbits represent the most commonly used laboratory strain owing to fewer health concerns [109]. As the cardiovascular system of the rabbit shares structural similarities with that of humans, they have been used in cardiovascular research [110]. Their overarching advantages, such as a unique feature of lipoprotein metabolism and similarities of eye size and bone metabolism, have resulted in their more frequent use as animal models in human research on atherosclerosis, lipid metabolism, eye disease (cornea, retina), and joint and bone disease [111,112,113]. They are also used as models for tracheal and dental regeneration [98,109].

Especially, recent studies have shown that rabbits—closely resembling human anatomy and physiology—are good predictors of responses in humans for stem cell-based therapy. Human adipose-derived mesenchymal stem cells (AD-MSCs) and bone marrow MSCs (BM-MSCs) showed significant improvement in the retinal injury rabbit model and the chemical burn injury rabbit model, respectively [114,115]. Positive results for the treatment of experimentally induced chondral defects and bone regeneration were reported in rabbits treated with autologous MSCs [116].

#### 3.1.3. Dog

Dogs are another frequently used, “default non-rodent” animal model owing to the practicality of their use and extensive information available [117]. Beagles are small dogs that are convenient to use in research studies; they represent the most popular breed for preclinical safety testing of pharmaceuticals. The internal systems, organs, and muscles of dogs are also physiologically and clinically more similar to those of humans than those of mice [118]. Furthermore, the relatively small size of dogs makes them easy to handle during clinical observations and blood sampling [119]. Rapid aging in dogs (12–15 years) reduces the time of disease development, leading to a significant reduction in the clinical trial durations [120]. Dogs exhibit a high incidence of malignancies and are, therefore, regarded as suitable models to study the etiology and pathogenesis of the said malignancies [121].

Dogs can serve as an experimental animal model for cartilage repair in humans since they share an osteoarthritis pathology with humans. Several in vivo studies have utilized AD-MSCs and BM-MSCs for osteoarthritis therapies in dogs [122,123]. Dogs can be used to model echocardiography and cardiac magnetic resonance imaging techniques for monitoring a wide range of cardiac parameters. Using their features, Gandolfi et al. [124] reported improvement of cardiac function in dogs treated with cardiac stem cells.

#### 3.1.4. Pig

Minipigs represent another good model for biomedical research. Small minipigs require less space in intensively controlled facilities, unlike conventional farm pigs. They easily adapt to laboratory housing and are convenient for use in experiments because of their small body size and ease of collection of fluids, including blood and other body fluids [125]. A high degree of morphological and physiological similarities has been observed between pig and human organs [126]. Remarkably, their gastrointestinal tract anatomy and physiological characteristics, including intestinal pH values, resemble those of humans [127]. Minipigs represent a suitable experimental model to study metabolic patterns in humans because of the similarities in hepatic cytochrome P450 [127], which is not expressed in dogs [128]. Minipigs present an alternative to the dog model because of the animal care and legal issues associated with the latter. Therefore, the biological similarities between minipigs and humans and relatively their easy handling facilitate their use as a non-rodent laboratory species, as a substitute for the dog, particularly in the EU [129].

For the advancement of stem cell therapy, a suitable animal model is required for the translation of research results into clinical trials. This is a very important component of stem cell research because the limitations of clinical trials are the major hurdles for such studies. The pig model has been used in stem cell research from HSC transplantation to regenerative medicine, including heart, cartilage, and bone regeneration [124,130,131,132].

#### 3.1.5. NHP

NHPs are phylogenetically close to humans, and Old World monkeys such as *Macaca fascicularis* (cynomolgus monkeys) and *Macaca mulatta* (rhesus monkeys) and New World monkeys such as *Callithrix jacchus* (the common marmoset) are widely used in biomedical research [133,134,135,136] despite several disadvantages of using an NHP model including high operation and maintenance costs, safety requirements, and ethical issues [137]. Old and New World monkeys exhibit very different characteristics, even though both are classified as primates. Cynomolgus and rhesus monkeys have an average lifespan of 30–40 years [138,139]. Female and male cynomolgus monkeys attain sexual maturity at 46 months and 42–60 months of age, respectively. Female and male rhesus monkeys attain sexual maturity at 34–43 months and 38 months of age, respectively [140].

In contrast, the marmoset has a lifespan of approximately 20 years and attains sexual maturity at 15–24 months of age with relatively more offspring (1–3) per delivery [141,142]. The common marmoset has been attracting considerable attention in the biomedical field by its size, short lifespan, easy availability, easy maintenance, and closer relationship to humans [143]. Therefore, marmosets can potentially be used in neuroscience, aging and chronic diseases, immunity and autoimmune disease, reproductive biology, stem cell research, and regenerative studies [144]. Despite the advantages of the marmoset model, lack of historical databases and experience in marmoset research results in the use of Old World monkeys in general toxicity studies with the availability of a large background database [145,146].

As a human-like model, NHPs can generate complementary data that develop stem cell-based therapeutic interventions between small animal models to humans. For example, the NHP models of Parkinson’s disease have contributed to stem cell-based therapies through the autologous or allogeneic approaches by analyzing the potential to differentiate into the dopaminergic neurons and reinnervate the putamen [147]. The streptozotocin (STZ)-induced type 1 diabetes model in NHPs has also been developed for assessing the effects of stem cells [148]. This NHP model has many advantages allowing continuous measurement of glucose and other various biochemical parameters through the catheter system. In addition, clinically relevant doses and routes of stem cell administration may be tested using this NHP model for further clinical trials.

## 4. Experimental Applications of Immunodeficient Animals

### 4.1. Rat and Mouse

As stated previously, SCID rodents are widely used in current research, and the use of large animals has also increased recently (Table 1). Mashimo et al. [90] reported the development of SCID rats using zinc finger nuclease (ZFN)-induced gene targeting of the rat IL2RG locus, where orthologous mutations cause X-SCID in humans and mice. Samata et al. [149] compared the in vivo effects of human dopaminergic neurons in nude and SCID mice and showed that SCID mice exhibit less spontaneous recovery in comparison with the nude mice. Rigatti et al. [150] evaluated the effects of polyomaviruses in a *Rattus* population using SCID mice and demonstrated that mutations in FOXN1 and IL2RG are associated with T cell immunodeficiency. Besch-Williford et al. [151] reported the identification, pathogenesis, and transmission of a novel polyomavirus in SCID F344 rats with null PRKDC and IL2RG genes. Ohno et al. [152] revealed the major role of host immunity in determining the carrying capacity of *Hymenolepis diminuta* in the intestines of SCID rats. Tanaka et al. [153] were the first to detect rat polyomaviruses infection in a colony of X-SCID rats in Japan. Beldick et al. [154] demonstrated the suitability of SCID rats for generating a robust model of neonatal hypoxic-ischemic brain injury. Humanized NOD/SCID/Il2rg null mice have been used as a model for the analysis of human immunodeficiency virus type 1 pathogenesis transplanted with HSCs [155]. Human B cell differentiation has been restored in NOD-SCID mice [156], and the effects of HSC transplantation were analyzed for diabetes in NOD mice [157].

### 4.2. Rabbit

Rabbit SCID models have also been developed and used in various studies. Rabbits with X-SCID have been developed using the CRISPR/Cas9 system targeting IL2RG (Table 2). These X-SCID rabbits presented immunodeficient phenotypes, including T and B cell loss and hypoplasia of the thymus [98]. SCID rabbit models were also established and used for studying *Pneumocystis* pulmonary infections [158].

### 4.3. Dog

Dogs have also been used as large SCID models. Hartnett et al. [159] reported the ability of CD34+ bone marrow cells to reconstitute normal B and T cell function in a nonablated large-animal model of bone marrow transplantation (Table 3). Goldschmidt et al. [160] used X-linked SCID dogs to study the response to papillomavirus infections progressing to metastatic squamous cell carcinoma. Meek et al. [89] reported that the dog SCID model results in a block in V(D)J recombination. Verfuurden et al. [161] identified that SCID dogs caused by a RAG1 mutation showed strongly reduced levels of immunoglobulins and lymphocytes consistent with a human SCID. X1-SCID dogs were used to test the therapeutic benefits of the foamy virus vector administered, expressing the human IL2RG gene [162].

### 4.4. Pig

SCID pig models show great potential for application in various human studies. Importantly, several SCID pig models lacking T and B cells have already been developed to meet the requirement of large animal models with higher similarity with humans [94,99]. These characteristics make them a more suitable model for the development of stem cell-based therapeutic strategies for human diseases. Suzuki et al. [45] reported the development of knockout-combined immunodeficient pig models similar to human X-SCID-like IL2RG, in which pigs were cloned by serial somatic cell nuclear transfer from porcine fibroblasts, and the IL2RG gene was disrupted by genome editing (Table 4). SCID pigs can also be developed using the reporter-guided transcription activator-like effector nuclease (TALEN) system [96]. Huang et al. [163] established a SCID pig model by targeting porcine RAG1/2 genes using TALEN technology. These immunodeficient pigs can be used for preclinical research and bridge the gap between small animals and humans. The SCID pig model was also used to study the replication of the Porcine reproductive and respiratory syndrome virus [164]. Ewen et al. [165] reported the similarity between the SCID pig model and other SCID models. SCID pigs exhibit a T and B lymphocyte-negative and NK cell-positive phenotype, which is consistent with the phenotypic properties of SCID in other animal species. Spontaneous mutations in the Artemis gene generated a SCID pig model lacking T and B lymphocytes with functional NK cells [63]. Since NK cells in Artemis^−/−^ SCID pigs are functional, Boettcher et al. [166] mutagenized IL2rg in an Artemis ^−/−^ mutant cell line to deplete NK cells, resulting in that pigs lacked T, B, and NK cells.

### 4.5. NHP

Although rodents with various gene deletions are available and are used in research, limitations still exist due to differences in the structure, physiology, and endocrinology of these animals in comparison with humans. Along with the necessity of NHP recognized in biomedical research, Niu et al. [167] applied CRISPR/Cas9 system to induced simultaneous disruption of target genes (Ppar-γ and RAG1) in cynomolgus monkeys (Table 5). In addition to Old World monkeys, Central Institute for Experimental Animals (CIEA) developed IL2RG-knockout SCID marmosets by high-efficiency gene modification using ZFN and TALEN technologies [91]. This technology facilitated the generation of marmosets with immunodeficient phenotypes that could successfully grow into adults. In 2019, Kumita et al. [168] additionally confirmed the efficient generation of knockout marmoset embryo via CRISPR/Cas9 gene editing, leading to an advance in SCID generation.

## 5. Conclusions

**Figure 2:**
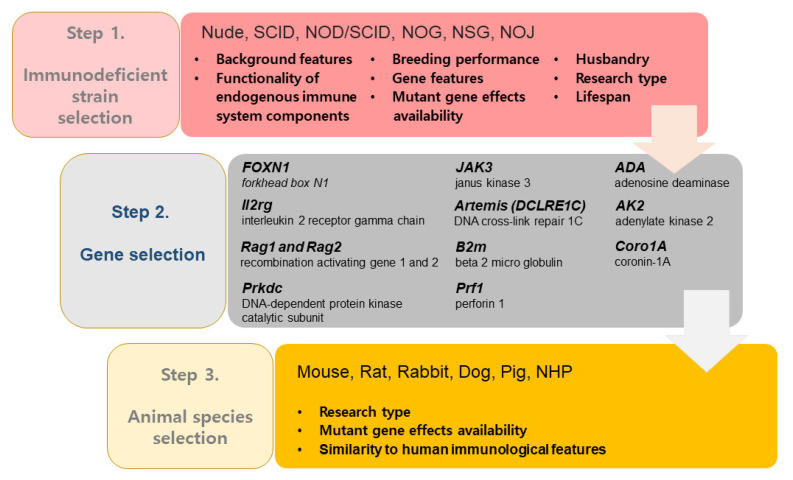
The process of proper immunodeficient animal selection for biomedical research.

In this review, we discussed the genetic background and utilization of the immunodeficient animals, including SCID animals, in various areas of biomedical research. Emerging therapeutic areas, such as stem cell therapy, xenotransplantation models, and humanized models, have necessitated the development of immunodeficient animal models, which were traditionally only used for preclinical cancer research. There are many different types of immunodeficient animal models, each with different genetic deficiencies, resulting in many different features in phenotype. Therefore, the most appropriate immunodeficient strain and target gene should be first selected based on the research type, gene feature, and mutant gene effects (Figure 2).

Many successful immunodeficient rodent models have been introduced in different research areas. Especially, humanized mice, defined as immunodeficient mice engrafted with human cells or tissues such as HSC, lymphoid tissue, or peripheral blood mononuclear cell [169], are the most commonly used model for studying human immune response and evaluating the efficacy of transplanted cells or tissues because of their easy breeding and handling and relatively low costs. However, phylogenetically closer large animal immunodeficient models with a better representation of human conditions are required due to genetic, anatomical, and physiological similarity to humans. The pig models can play a vital role, particularly in the field of xenotransplantation, due to the 80% similarity of pig’s immune system to human [67]. In addition, the NHPs can be the most accurate models for different preclinical studies due to the close phylogenetic relationship with humans. In addition, human longevity has increased, which has led to studies on inheritable genetic diseases and the development of regenerative medicine strategies. This necessitates large animal immunodeficient models with the advent of various next-generation therapies, such as chimeric antigen receptor (CAR) T-cell, stem cell therapy, and tissue engineering, although a major concern of utilizing large animals is the cost and limited supply due to animal ethics and social constraints.

As an accessible and appropriate large animal model for immunodeficiency, different SCID pig and NHP models have already been established. Especially, recent advances in the genome-editing technique such as the CRISPR/Cas system have made it possible to generate gene-modified SCID NHP models, Old World monkeys, and New World monkeys. According to research areas, it will be important to select the optimal large animal models with consideration of various gene effects that can control T cell, B cell, or NK cells associated with innate and adaptive immunity after studies using small animal models. Taken together, we believe that large animal SCID models using pigs and NHPs will emerge as popular animal models for regenerative medicine, clinical research, and understanding human immune diseases and physiology between small animal models and humans.

## Figures and Tables

**Table 1:** Studies using severe combined immunodeficient (SCID) mice and rats and mice as the animal model.

Deleted Genes	Description	References
IL2rg	Generation of knockout rats with X-SCID using zinc-finger nucleases	[90]
IL2rg	Assessing in vivo function of human dopaminergic neurons using X-SCID rats	[149]
IL2rg	Evaluation of T-cell immune surveillance, critical for commensal polyomavirus control, in SCID rats	[150]
Prkdc, IL2rg	Identification, pathogenesis, and transmission of a novel polyomavirus in SCID F344 rats with null Prkdc and IL2rg genes	[151]
IL2rg	Identification of a major role for host immunity in determining the carrying capacity of *H. diminuta* in intestines of SCID rats	[152]
IL2rg	Detection of the rat polyomaviruses infection in a colony of X-SCID rats	[153]
Prkdc	Effects of hiPSC-NPCs in SCID rats as a model of neonatal hypoxic-ischemic brain injury	[154]
IL2rg	Analysis of human immunodeficiency virus type 1 pathogenesis in humanized NOD/SCID/Il2rg null mice transplanted with HSCs	[155]
Rag1	Restoration of human B-cell differentiation into NOD-SCID mice	[156]

**Table 2:** Studies using SCID rabbit model.

Deleted Genes	Description	References
IL2rg	Development and maintenance of stable strains of rabbits with X-SCID via the CRISPR/Cas9 system targeting Il2rg	[98]
IL2rg	Establishment of SCID rabbit models for the development of early diagnostics and therapeutics for immunodeficient patients	[158]

**Table 3:** Studies using SCID dogs.

Deleted Gene	Description	References
IL2rg	Demonstration of CD34+ bone marrow cells to reconstitute normal B- and T-cell function in X-SCID dogs	[159]
IL2rg	Analysis of the response to papillomavirus infections progressing to metastatic squamous cell carcinoma.	[160]
Prkdc	Comparison of SCID animal models on the severity of the V(D)J recombination defects	[89]
Rag1	Analysis of SCID dogs with Rag1 mutation	[161]
IL2rg	Intravenous injection of a foamy virus vector expressing the human IL2RG gene for the correction of SCID-X1 dogs	[162]

**Table 4:** Studies using SCID pigs.

Deleted Genes	Description	References
IL2rg	Evaluation of preclinical regenerative stem cell strategies for clinical therapy	[45]
Rag2	Demonstration of the growth of mature teratomas from human pluripotent stem cells in SCID pigs	[96]
Rag1/2	Establishment of a SCID pig model by targeting porcine RAG1/2 genes via TALEN technology	[163]
IL2rg	Elucidation of the SCID phenotype by enumerating circulating white blood cell populations	[165]
Artemis	Generation of SCID pig model by spontaneous mutations in the Artemis gene	[63]
Artemis, IL2rg	Use of Artemis and IL2rg for SCID pigs lacking T, B, and NK cells	[166]

**Table 5:** Studies using SCID nonhuman primates (NHPs).

Species/Strain	Deleted Genes	Description	Reference
NHP/cynomolgus	Rag1	Generation of gene-modified cynomolgus monkey via CRISPR/Cas9 system	[167]
NHP/marmoset	IL2rg	Development and evaluation of IL2rg knockout marmosets with immunodeficient phenotypes, possible to grow to adults	[91]

## Data Availability

Data available in a publicly accessible repository.
